# Estimating the organic oxygen content of biochar

**DOI:** 10.1038/s41598-020-69798-y

**Published:** 2020-08-04

**Authors:** Santanu Bakshi, Chumki Banik, David A. Laird

**Affiliations:** 1 Department of Agronomy, Iowa State University, Ames, IA 50011 USA; 2 Present Address: 3118 Biorenewables Research Laboratory, Bioeconomy Institute, Iowa State University, Ames, IA 50011 USA; 3 Present Address: Department of Agricultural and Biosystems Engineering, Iowa State University, Ames, IA 50011 USA

**Keywords:** Climate sciences, Environmental sciences, Chemistry, Energy science and technology

## Abstract

The organic O content of biochar is useful for assessing biochar stability and reactivity. However, accurately determining the organic O content of biochar is difficult. Biochar contains both organic and inorganic forms of O, and some of the organic O is converted to inorganic O (e.g., newly formed carbonates) when samples are ashed. Here, we compare estimates of the O content for biochars produced from pure compounds (little or no ash), acid-washed biomass (little ash), and unwashed biomass (range of ash content). Novelty of this study includes a new method to predict organic O content of biochar using three easily measured biochar parameters- pyrolysis temperature, H/C molar ratio, and %biochar yield, and evidence indicating that the conventional difference method may substantially underestimate the organic O in biochar and adversely impact the accuracy of O:C ratios and van Krevelen plots. We also present evidence that acid washing removed 17% of the structural O from biochars and significantly changes O/C ratios. Environmental modelers are encouraged to use biochar H:C ratios.

## Introduction

Interest in the use of biochar as a soil amendment to enhance soil quality and sequester C has increased substantially during the last decade. Soil biochar applications are effective for C-sequestration because photosynthesis transfers C from the atmosphere to biomass and pyrolysis transforms some of the biologically labile biomass C into recalcitrant biochar C, which can stay in the soil for hundreds to thousands of years^1–4^. Soil biochar applications are also of interest because of their potential to positively impact soil quality and crop productivity, particularly when applied to degraded and otherwise problematic soils.

The impact of soil biochar applications on C sequestration and various agronomic and environmental outcomes is influenced by the physical and chemical properties of the biochar. Amonette and Joseph^5^ reported that the elemental content of biochar depends on the biomass feedstock whereas pyrolysis temperatures has a large influence on functional group and structural chemistry of biochars. The pyrolysis process favors the elimination of H and O over C from the organic phase and increasing the pyrolysis temperature drives the elimination of H and O towards completion^6–8^. However, increasing the pyrolysis temperatures also promotes the formation of inorganic phases, particularly carbonates, which contain both O and C. Carbon is also the primary element in condensed aromatic structures, which dominate the organic phase of biochar; while O is the key element in many polar organic functional groups on biochar surfaces, which influence biochar reactivity in soil environments. Thus, O and C are constituents of both the organic and inorganic phases in most biochar samples.

Knowing the O:C elemental ratio of the organic phase apart from the O:C ratio of the whole biochar is important for many reasons. The O:C ratio of the organic phase is a measure of the density of polar functional groups on biochar surfaces. Cheng et al.^9^ and Crombie et al.^10^ mentioned the importance of O to C ratio of fresh biochars as an index for the extent of charring and the O:C ratio can also be used to assess the extent of post-pyrolysis oxidation, which occurs as biochars age/weather in soil environments^11^. Masiello^12^ discussed the potential use of O:C ratios for assessing biochar stability in soils. Spokas^13^ noted that most biochars have O:C ratios within 0.2–0.6 range with O:C ratios approaching 0.6 indicating the least stable biochars. The International Biochar Initiative (IBI) reported various alpha methods including the use of O:C ratio^14^ to assess biochar stability and biochar degradation patterns.

Various analytical methods have been used to determine the O content of biochars. For instance, the O content of biochar has been determined by difference from unity after subtracting the ash content and elemental C, H, N and S content as determined by the proximate and ultimate analysis methods^15,16^, respectively. Numerous studies ^17-22^ have used the difference method for calculating the O content of biochars. However, the difference method only works for low ash biochars and biochars that contain SiO_2_ as the only significant form of ash.

The presence of alkali and alkaline earth metals (e.g., K, Ca, and Mg) in biochar is problematic for determination of O by the difference method, because these metals may be associated with carboxylate functional groups, which are part of the organic phase in biochar, or halides admixed with the biochar but will be associated with carbonates after the biochar sample is ashed^23^. New carbonate anions are produced during ashing from O and C atoms that were part of the organic phase of the biochar; and, the presence of newly formed carbonates in the ash will result in underestimation of the organic O content of biochars when the difference method is used.

A second method for determining organic O content and O:C ratios of the organic phase is to first treat biochar samples with acid and then use the difference method described above. Acid pre-treatments remove carbonates and alkali and alkaline earth metals from the biochar sample; and Si, which is not removed by acid treatments, can be assumed to have SiO_2_ stoichiometry. Hence, this approach gives accurate estimates of the organic O and O:C ratios of acid pre-treated biochars. However, acid pre-treatments can alter the surface chemistry of biochars, removing some O containing functional groups from biochar surfaces^23^.

A third method of determining the O content of the organic phase of biochars is to use total chemical analysis and an assumed inorganic stoichiometry to estimate the inorganic O content and then subtract inorganic O from total O. This third approach is also problematic, because the ash that is admixed with biochar may include carbonate, halide, oxide and/or hydroxide phases^24^ and some of the alkali and alkaline earth metals may be associate with carboxylate groups that are part of the biochar organic phase. As a result, the stoichiometry of the inorganic phases in biochar ash is difficult or impossible to accurately determine.

The determination of O content of the organic phase of biochars has until now been problematic because of the problems described above. For example, Ronsse et al.^25^ intentionally avoided measuring or calculate the O content of biochars, while Lawrinenko and Laird^26^ were able to determine the O content of biochars produced from cellulose, which contains negligible ash, but were not able to determine the O content of biochars produced from maize stover, alfalfa or albumin, all of which contain significant amounts of ash. Therefore, a method for accurate determination of the O content of the organic phase of biochar is needed to enhance understanding of biochar surface chemistry, weathering transformations, and stability in soil environments. Our overall objective was to develop a more reliable method to estimate the O content of the organic phase of biochar and assess the magnitude of error associated with conventional difference methods of determining the O content of biochars. The specific objective of this study was to evaluate the relationship between the O content of biochars produced from pure compounds, which contain little or no ash, and pyrolysis temperature, biochar yield and H:C ratios to determine whether these easily measure biochar properties can be used to estimate O:C ratios and the O content of the organic phase of biochars produced from biomass.

## Results and discussion

### Elemental analysis of pure compounds and pure compound biochars

The mass yield of biochars is known to decrease with increasing pyrolysis temperature^27-29^. In our study, biochar mass yields for tetracycline, glycine, vitamin C, and cellulose all decreased systematically with increasing pyrolysis temperature (Fig. S1). By contrast, the biochar mass yield for polyethylene glycol decreased abruptly between 300 and 400 °C and was very low for all pyrolysis temperatures above 300 °C. The abrupt decrease in mass yield for polyethylene glycol biochar is attributed to thermal depolymerization and volatilization loss of monomeric and oligomeric compounds for pyrolysis temperatures above 300 °C. Because our results indicate that polyethylene glycol depolymerized and volatilized rather than pyrolyzing, we did not use the biochars prepared from polyethylene glycol in our analysis. In our study, tetracycline had the highest biochar mass yield for all pyrolysis temperatures (300 to 900 °C), due to its high molecular weight and the presence of aromatic structures in tetracycline. Cellulose had the lowest biochar mass yields (excluding polyethylene glycol) for all studied pyrolysis temperatures. Cellulose has no aromatic C and a high concentration of structural OH groups, which are easily volatilized during pyrolysis.

The structural chemistry of feedstocks influences the mass yield and elemental composition of biochars. Oxygen is associated with functional groups in biomass feedstocks (hydroxyls, phenols, ethers, carbonyls, and carboxyls); while H is associated with surface functional groups, aliphatic compounds, and the surfaces of aromatic structures (aromatic C-H). During pyrolysis the structural H and O are lost primarily as H_2_O while C is condensed into aromatic structures^30–34^. In our study, the elemental composition of the pure compound biochars varied systematically with pyrolysis temperature. The C content of the pure compound biochars increased linear with pyrolysis temperature (R^2^ > 0.93, *P* < 0.05: Fig. S2a). By contrast, both the O content and H content of the pure compound biochars decreased with increasing pyrolysis temperature (Fig. S2b and S2c) due to selective volatilization of these elements. The systematic changes in biochar elemental composition are also reflected in the inverse relationships between H/C and O/C molar ratios and pyrolysis temperature (Fig. S3a and S3b). Our results, however, demonstrate systematic changes in elemental compositions with increasing pyrolysis temperatures that are unique for each of the biochars produced from the pure compounds.

**Figure 1:**
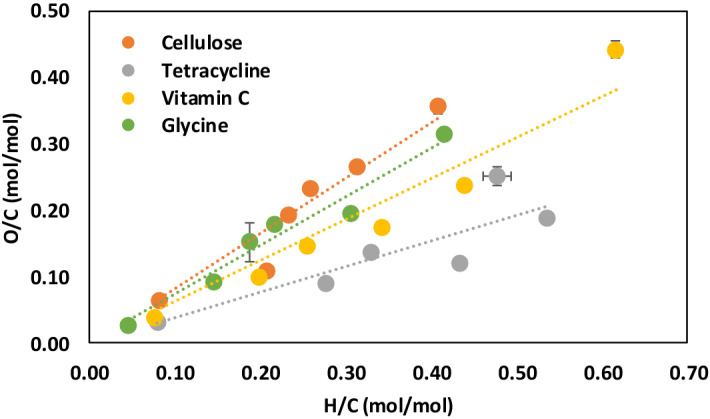
van Krevelen plot showing the relationship between H/C and O/C molar ratios for pure compound biochars produced at peak pyrolysis temperatures ranging from 300 to 900 °C. Slopes of the H/C vs. O/C relationships depend on properties (chemistry) of the feedstock. Symbols showing standard error for sample size, n = 3.

Van Krevelen plots, showing the relationship between H/C and O/C molar ratios of biochars, have been widely used to characterize the extent of pyrolysis^35-37^. We found significant linear correlations (R^2^ > 0.95) between H/C and O/C molar ratios for the pure compound biochars (Fig. 1). Our results for the pure compound biochars demonstrate that different feedstocks have unique H/C vs O/C slopes, which are characteristic of the feedstock properties.

**Figure 2:**
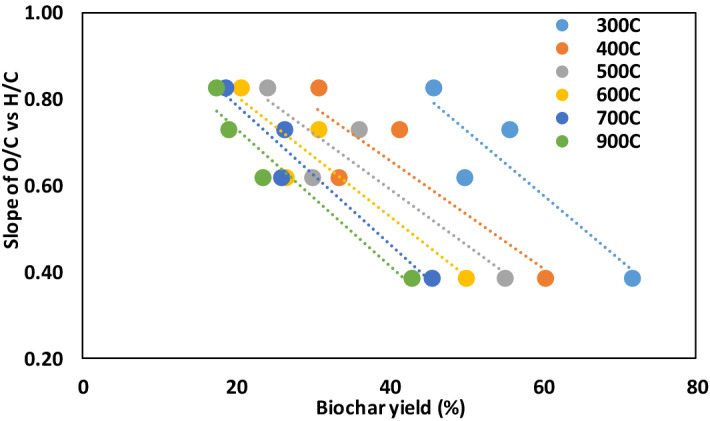
Slopes of the O/C vs. H/C relationships are inversely related to biochar yield for peak pyrolysis temperatures ranging from 300 to 900 °C. Slopes of the O/C vs. H/C relationships are nearly independent of peak pyrolysis temperature while the intercept decreases with increasing pyrolysis temperature.

Recognition that the slope of the H/C vs. O/C relationship is unique for biochars prepared from different feedstocks allow us to develop a model for predicting the organic O (oO) and O/C molar ratios of biochars. As a first step, we used regression analysis forcing the intercept through zero to determine the slopes of the H/C vs. O/C relationships for the pure compound biochars (Fig. 1). In the second step, we found negative linear correlations between the slopes of the H/C vs. O/C relationships and biochar mass yield for all pyrolysis temperatures (Fig. 2, R^2^ = 0.79–0.93; *P* < 0.05). The slopes of these linear relationships are similar for all pyrolysis temperatures (between − 0.013 and − 0.016) and become increasingly linear with increasing pyrolysis temperature. This result indicates that the biochar mass yield at a specific pyrolysis temperature, which is easily measured, can be used to predict the slope of the H/C vs. O/C relationship for a specific feedstock. Based on this analysis we propose that the slope of the H/C vs. O/C relationship for a specific feedstock is a linear function of the biochar mass yield at a specific pyrolysis temperature (Eq. 1);

1 $$Slope\, of\, H/C \;vs.\; O/C = A(biochar\, yield) + B$$

where A and B are constants for a specific peak pyrolysis temperature.

As an initial test of our approach, we derived an equation (Eq. 2) for predicting molar O/C ratios of the pure compound biochars that uses biochar mass yields for 500 °C peak pyrolysis temperature (e.g., *yield*500) and measured molar H/C ratios. The values of the constants in Eq. 2 were determined by optimizing the fit between the measured and predicted O/C ratios for the pure compound biochars.

2 $$\frac{O}{C}= -0.02155+[\left\{-0.0129 \left(yield500\right)+1.1095\right\}\left(\frac{H}{C}\right)]$$

By multiplying both sides of Eq. 1 by the mass of C in the biochar (%C) and the atomic weight of O and dividing by the atomic weight of C an equation predicting the mass percent of oO (%oO) in the pure compound biochars is obtained (Eq. 3):

3 $$\%oO=[-0.02155+\left[\left\{-0.0129 \left(yield500\right)+1.095\right\}\left(\frac{H}{C}\right)\right]]\times \%C \times \left(\frac{16}{12}\right)$$

Similar equations can be developed for other pyrolysis temperatures, but here we considered only the equation for pyrolysis at 500 °C.

**Figure 3:**
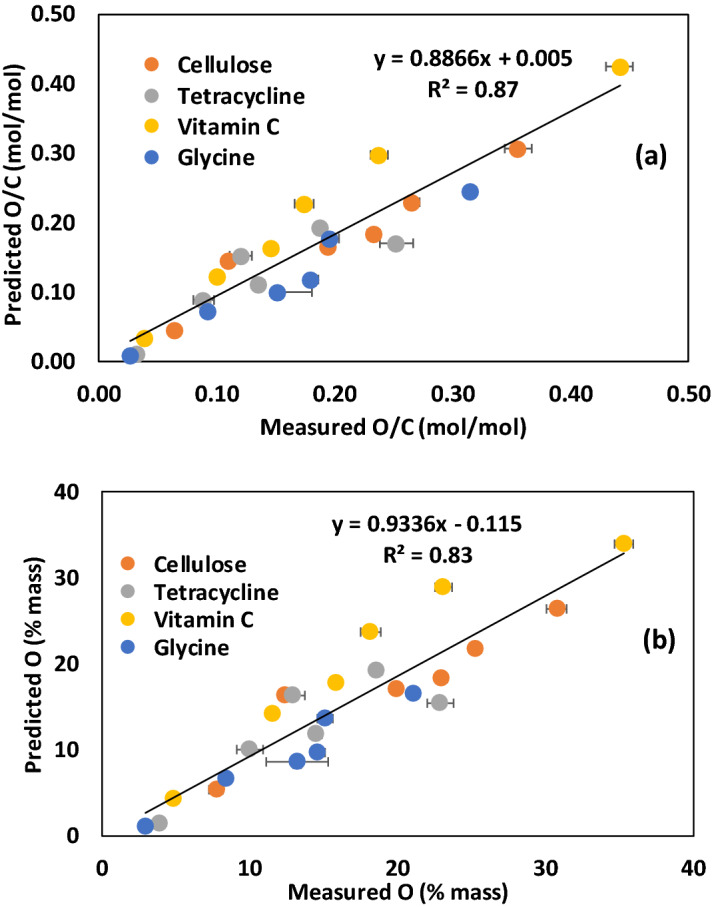
Relationships between measured and predicted a) O/C molar ratios and b) mass percent O for the pure compound biochars produced at peak pyrolysis temperatures ranging from 300 to 900 °C. Measured O (mass %) was determined by the conventional difference method, predicted O/C molar ratios were determined using Eq. 2, and predicted O (mass %) was determined using Eq. 3. Symbols showing standard error for sample size, n = 3.

Equation 2 predicts the O/C molar ratios of the four pure compounds (Fig. 3a, R^2^ = 0.87; *P* < 0.05) and Eq. 3 predicts the %oO of the four pure compound biochars (Fig. 3b, R^2^ = 0.83; *P* < 0.05) with reasonable accuracy relative to measured O/C and %oO values where %oO was determined by the conventional difference method. Here we assumed that the inorganic O content (%iO) of the biochars was zero, because the pure compounds contained negligible levels of ash. This finding is consistent with our hypothesis that the degree of biochar aromaticity and hence the O/C and H/C molar ratios of biochar depend on the peak pyrolysis temperature and the composition of the biomass feedstock used to produce the biochar.

Equations 2 and 3 offer three potential advantages for estimating O/C and %oO of biochars. (1) This approach does not rely on %oO determined by the difference method, which, as discussed in the introduction section, is problematic for biochar samples that contain alkali and alkaline earth metals. (2) This approach can be used with biochars produced from feedstocks of contrasting chemical composition. And, (3) the approach requires only four measured parameters (%C, %H, %N, and %biochar yield at specific pyrolysis temperature) all of which can be easily and accurately measured. A major limitation of Eqs. 2 and 3 is that as derived they are only valid for pure compound biochars.

### Model development

We suggest that Eqs. 2 and 3 derived above for biochars produced from pure compounds that contain negligible ash can be modified to predict the %oO and O/C molar ratios for biochars produced from biomass that contain ash. Key to application of Eqs. 2 and 3 with biochar samples derived from biomass is recognition that the biochar yield term (“y*ield*500” in Eqs. 2 and 3) is the ash free yield (A*yield*500) not the total yield of biochar from the pyrolysis of biomass, where:

4 $$Ayield500=yield500\frac{\left( \%C+\%N+\%H+\%S+\%oO\right)}{100}$$

Equation 4 is predicated on the assumption that the only elements in the organic phase of the biomass biochars are C, N, H, S and organic O. Carbon, N, H, S, and oO are the only elements in the pure compound biochars, discussed above, and therefore A*yield*500 = *yield*500 for the pure compound biochars. For biomass biochars, which contain ash, A*yield*500 will be less than *yield*500 by the amount of ash that is actually in the biochar sample. However, it is important to recognize that the amount of ash in a biochar sample does not equal the amount of ash that is determined by ashing the sample in a muffle furnace because new carbonates and oxides are formed during the ashing process^11^.

By combining Eqs. 2 and 3 with Eq. 4 we are able to determine %oO and O/C molar ratio. Thermal combustion analyzers are able to readily quantify %C, %N, %H, and %S in a biochar sample, but not %oO. For simplicity, we define the term ‘Sum’ as:

5 $$Sum=\%C+\%N+\%H+\%S$$

Therefore, Eq. 2 can be rewritten for a biomass biochar as follows:

6 $$\frac{O}{C}= -0.02155+[\left\{-0.0129 \left(Ayield500\right)+1.1095\right\}\left(\frac{H}{C}\right)]$$

7 $$\frac{O}{C}= -0.02155+[\left\{-0.0129 \left(yield500\frac{\left( Sum +\%oO\right)}{100} \right)+1.1095\right\}\left(\frac{H}{C}\right)]$$

8 $$\frac{O}{C}= -0.02155-\left\{0.000129 \times \left(yield500\right) \times Sum \times \left(\frac{H}{C}\right)\right\}-\left\{0.000129\times \left(yield500\right) \times \%oO \times \left(\frac{H}{C}\right)\right\}+\left\{1.1095 \times \left(\frac{H}{C}\right)\right\}$$

And recognizing that $\%oO=\{\left(\frac{O}{C}\right)\times \%C \times \left(\frac{16}{12}\right)\}$ , where 12 and 16 are the atomic weights of C and O, respectively, we obtain:

9 $$\frac{O}{C}= -0.02155-\left\{0.000129 \times \left(yield500\right) \times Sum \times \left(\frac{H}{C}\right)\right\}-\left\{0.000129\times \left(yield500\right) \times \%\left(\frac{O}{C}\right)\times \%C \times \left(\frac{16}{12}\right) \times \left(\frac{H}{C}\right)\right\}+\left\{1.1095 \times \left(\frac{H}{C}\right)\right\}$$

Rearranging Eq. 9 we obtain the Eq. 10, which can be used to determine the molar O/C ratio of the organic phase in a biochar sample:

10 $$\frac{O}{C}=\frac{\left[-0.02155-\left\{0.000129 \times \left(yield500\right) \times Sum \times \left(\frac{H}{C}\right)\right\}+\left\{1.1095 \times \left(\frac{H}{C}\right)\right\}\right]}{[1+\{0.000172 \times \left(yield500\right) \times \%C \times \left(\frac{H}{C}\right)]} $$

By multiplying both sides of Eq. 10 by the mass of C in the biochar (%C) and the atomic weight of O and dividing by the atomic weight of C an equation predicting the mass percent of organic O (%oO) is obtained (Eq. 11):

11 $$\%oO= \frac{\left[-0.02155-\left\{0.000129 \times \left(yield500\right) \times Sum \times \left(\frac{H}{C}\right)\right\}+\left\{1.1095 \times \left(\frac{H}{C}\right)\right\}\right]}{[1+\{0.000172 \times \left(yield500\right) \times \%C \times \left(\frac{H}{C}\right)]} \times \%C \times \left(\frac{16}{12}\right)$$

### Analysis of acid washed biochars from biomass feedstocks

The mass yield and elemental composition of the acid (0.05 M HCl) washed biochars produced at 500 °C from different biomass feedstocks (RO = red oak, AM = alfalfa meal, SG = switchgrass, CS = corn stover, LP = loblolly pine, and SS = soybean stover) are presented in Table 1. The %ash determined by ashing the acid washed biochar at 730 °C ranged from 0.96% to 15.86%. Silica, assuming SiO_2_ stoichiometry, explained 18.7 to 78.8% of the measured ash mass of the acid washed biochars. Other elements present in the ash are C, H, N, S, Na, K, Ca, Mg, Mn, Cu, P, Al, Zn, Fe, and Sr. The small amount of C in the ash of the acid washed biochars (< 3% of ash mass) is attributed to small amounts of carbonates and/or graphitic C that formed during the ashing of the biochars. Although heavy metals and alkali and alkaline earth metals were detected in the ash of the acid washed biochars these elements represented less than 1% of the mass of the acid washed biochars. The sum of all elements measured in the ash samples explained 62.42 to 98.35% of the measured ash mass for the acid washed biochars (Table 2). Hence, the amount of iO (other than O in SiO_2_) in the acid washed biochars is < 0.5% of the biochar mass.

**Table 1:** Physico-chemical properties of 0.05 M HCl treated biochars from biomass feedstocks studied here.

Biochar	Yield (%)	C (%)	N (%)	H (%)	S (%)	Si (%)	O (%)			Ash (%)
							Inorganic O: obtained from SiO_2_ stoichiometry	Organic O: obtained from by difference	Predicted organic O	
RO	23.97 ± 0.11	82.27 ± 0.4	0.005 ± 0.0005	3 ± 0.01	0.07 ± 0.02	0.165 ± 0.02	0.19 ± 0.03	14.3 ± 0.01	16.44 ± 0.05	0.3866
AM	24.94 ± 0.21	67.49 ± 0.64	3.94 ± 0.04	3.16 ± 0.05	0.38 ± 0.08	4.43 ± 0.07	5.04 ± 0.08	15.55 ± 0.05	18.16 ± 0.76	2.8407
SG	24.2 ± 0.22	70.6 ± 0.68	1.23 ± 0.02	3.12 ± 0.06	0.41 ± 0.07	4.52 ± 0.08	5.15 ± 0.09	14.97 ± 0.1	18.06 ± 0.55	1.0397
CS	24.05 ± 0.4	76.97 ± 0.37	1.2 ± 0.009	3.1 ± 0.03	0.08 ± 0.006	1.64 ± 0.04	1.87 ± 0.05	15.14 ± 0.01	17.38 ± 0.56	0.5194
LP	29.05 ± 0.3	83.82 ± 0.2	0.001 ± 0.0009	3.08 ± 0.009	0.16 ± 0.03	0.09 ± 0.005	0.104 ± 0.007	12.73 ± 0.08	15.15 ± 0.26	0.5058
SS	23.12 ± 0.11	75.63 ± 0.42	1.27 ± 0.02	2.9 ± 0.07	0.73 ± 0.44	2.51 ± 0.05	2.86 ± 0.05	14.09 ± 0.002	16.6 ± 0.95	2.2354

**Table 2:** Composition of ash from the 0.05 M HCl treated biomass biochars relative to the measured mass of the ash.

Biochar	CHNS (%)	Inorganic elements (%)	SiO_2_ (%)	Total elements (%)
RO	2.88 ± 0.02	40.27 ± 1.3	36.99 ± 1.25	77.27
AM	1.3 ± 0.005	19.53 ± 2.28	78.82 ± 2.05	98.35
SG	0.58 ± 0.01	6.56 ± 1.1	61.01 ± 0.5	67.57
CS	0.83 ± 0.06	8.05 ± 2.9	54.36 ± 0.5	62.42
LP	1.02 ± 0.009	48.64 ± 1.05	18.7 ± 0.08	67.33
SS	0.8 ± 0.07	28.48 ± 0.8	68.43 ± 1.02	96.91

Elements in ash include C, H, N, and S as measured by CHNS analyzer; Na, K, Ca, Mg, Mn, Cu, P, Al, Zn, Fe, and Sr as measured by ICP-OES after acid digestion; and SiO_2_ as calculated from Table 1 stoichiometry.

We tested Eqs. 10 and 11 by analyzing biochars prepared from acid washed RO, AM, SG, CS, LP, and SS biomass feedstocks. Equation 10 was used to estimate the O/C molar ratio of the acid washed biomass biochars and compared with the measured O/C molar ratios as determined by a modified difference method (Eq. 12;

12 $$\frac{O}{C}=\frac{\left\{\left(100-\%C-\%H-\%N-\%S-\%Si-\%iO\right) \times \left(\frac{12}{16}\right)\right\}}{\%C}$$

where %Si was determined by ashing the acid washed biochars at 730 °C and quantifying Si by ICP-OES. The %iO was calculated from the measured %Si assuming SiO_2_ stoichiometry (Eq. 13);

13 $$\%iO=\left\{\left(\frac{\%Si\, in\, ash\, \times \%ash\, in\, biochar}{100}\right) \times \left(\frac{2 \times 16}{28}\right)\right\}$$

where 16 and 28 are the atomic weights of O and Si, respectively. Using Eq. 13 to calculate %iO, the ‘measured’ %oO in the acid washed biochars can be calculated by the following equation:

14 $$\%oO=100-\%C-\%N-\%H-\%S- \left(\frac{\%Si\, in\, ash\, \times \%ash\, in\, biochar}{100}\right)-\left\{\left(\frac{\%Si\, in\, ash\, \times \%ash\, in\, biochar}{100}\right) \times \left(\frac{2 \times 16}{28}\right)\right\}$$

**Figure 4:**
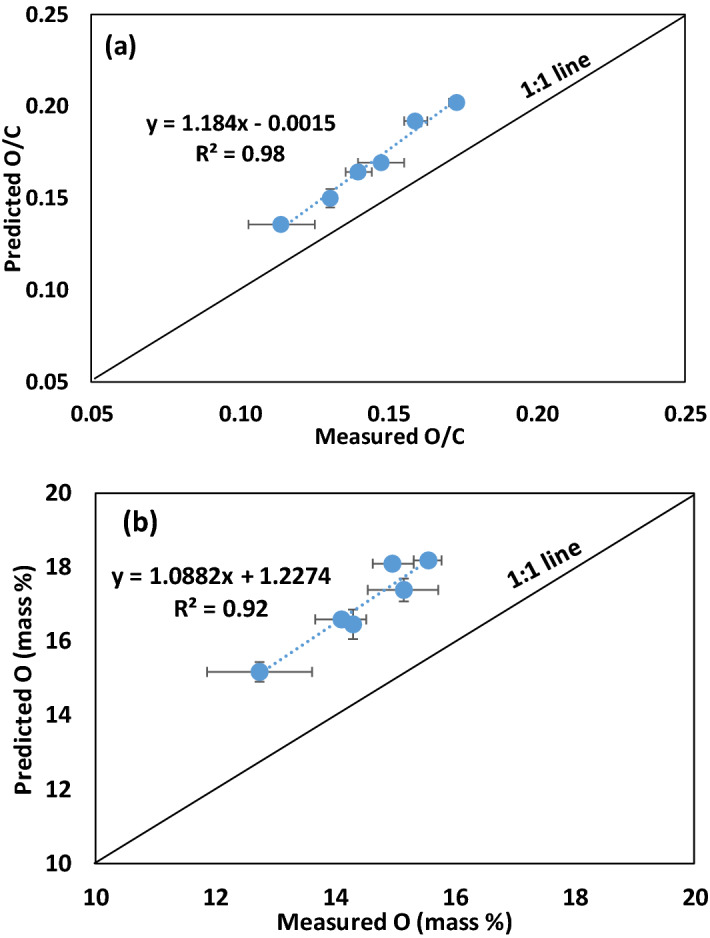
Relationships between measured and predicted a) O/C molar ratios and b) mass percent O for the acid washed biochars produced from pyrolysis of biomass at 500 °C. Measured O (mass %) was determined by the modified difference method, measured O/C molar ratios were determined by dividing %oO by %C and adjusting for atomic weights, predicted O/C molar ratios were determined using Eq. 10, and predicted oO (mass %) was determined using Eq. 11. Symbols showing standard error for sample size, n = 3.

We found strong linear correlation between the predicted O/C (using Eq. 10) and measured O/C (using Eq. 12) (Fig. 4a, R^2^ = 0.98; *P* < 0.05). However, the predicted O/C molar ratios average 17.3 ± 2.3% higher than the measured O/C molar ratios determined by the modified difference method (Eq. 12). The correlation between predicted %oO (using Eq. 11) and measured %oO (Eq. 14) was also strong (Fig. 4b, R^2^ = 0.92; *P* > 0.05). However, the predicted %oO also average 17.3 ± 2.3% higher than the measured %oO determined by the modified difference method. The strong linear correlation between measured and predicted O/C molar ratios and %oO for the acid washed biochars supports the validity of our model (Eqs. 10 and 11). The 17.3% discrepancy between measured and predicted O/C molar ratios and %oO content of the acid washed biochars is attributed to the removal of some of the organic O functional groups during the acid washing treatments. Mass yields of the acid washed biochars (Table 1) ranged from 1.6 to 6.7% lower than the mass yields of the untreated biomass biochars (Table 3) due to the removal of salts, carbonates, acid soluble organic compounds (often rich in O), and various acid catalyzed dehydration reactions during the acid wash treatments. Acids are widely recognized for causing dehydration reactions such as converting alcohols to ethers and alkenes with the loss of a structural O as H_2_O. Acids may also accelerate some decarboxylation reaction, which result in the loss of a structural O as CO_2_^38^. Thus, acid washing of the biochars is anticipated to remove some structural O from biochars.

**Table 3:** Physico-chemical properties of untreated biochars from biomass feedstocks studied here.

Biochar	Yield (%)	C (%)	N (%)	H (%)	S (%)	Ash (%)	O (%)	
							By difference	**Predicted**
RO	26.36	81.55 ± 0.49	0.006 ± 0.001	3.124 ± 0.04	0.136 ± 0.03	1.052 ± 0.09	14.13 ± 0.56	16.75 ± 0.215
AM	29.89	57.96 ± 0.09	3.56 ± 0.01	3.24 ± 0.02	0.25 ± 0.009	21.5 ± 0.08	13.51 ± 0.18	18.69 ± 0.07
SG	30.91	66.02 ± 0.64	1.204 ± 0.007	3.37 ± 0.06	0.094 ± 0.005	17.3 ± 0.06	12 ± 0.6	18.46 ± 0.43
CS	26.52	73.66 ± 0.82	1.07 ± 0.04	3.09 ± 0.01	0.06 ± 0.005	10.35 ± 0.125	11.77 ± 0.92	17.29 ± 0.08
LP	30.65	84.73 ± 0.46	0.002 ± 0.005	3.11 ± 0.08	0.09 ± 0.009	1.36 ± 0.11	10.71 ± 0.39	15.04 ± 0.5
SS	26.2	65.69 ± 0.77	1.145 ± 0.03	3.11 ± 0.03	0.07 ± 0.004	16.07 ± 0.04	13.91 ± 0.82	18.29 ± 0.12

We tested the effect of acid washing on the structural O content of the biochars prepared from tetracycline. The oO content of the acid (0.05 M HCl) washed tetracycline biochar (500 °C) was 11.93% lower than the oO content of the untreated tetracycline (500 °C) biochar; hence the loss of oO during acid washing treatment explains much of 17.3% difference between the measured and predicted %oO content of the acid washed biomass biochars in Fig. 4b.

The results for the acid washed biochars suggest four important conclusions: (1) Our model (Eqs. 10 and 11) is valid but only for fresh biochars. (2) Acid washing removes some but not all O containing surface functional groups from biochars (aging and other treatments, such as H_2_O_2_ or washing with base, may add new O containing functional groups onto the surfaces of biochar). (3) The modified difference method, which includes subtracting measured %C, %N, %H, %S, %Si, and %iO (assuming SiO_2_ stoichiometry) from 100, should be used to determine the %oO content of acid washed biochars. And 4) our model (Eqs. 10 and 11) over predicts %oO and O/C molar ratios for the acid washed biochars, because of the loss of structural O during the acid washing treatment. However, Eqs. 10 and 11 could be easily recalibrated to predict %oO and O/C molar ratios of acid washed biochars.

### Analysis of untreated biochars produced from biomass feedstocks

The elemental composition and ash content of the untreated biomass biochars are presented in Table 3. The H/C molar ratios for herbaceous biochars are higher than the H/C molar ratios of biochars produced from woody biomass, which suggests that the woody biochars are more aromatic than the herbaceous biochars produced at the same temperature. This observation is attributed to the higher cellulose and hemicellulose and lower lignin content of herbaceous biomass relative to woody biomass^39^. The herbaceous biochars also have higher ash content of than wood biochars, which is consistent with previous research^11,40,41^.

**Figure 5:**
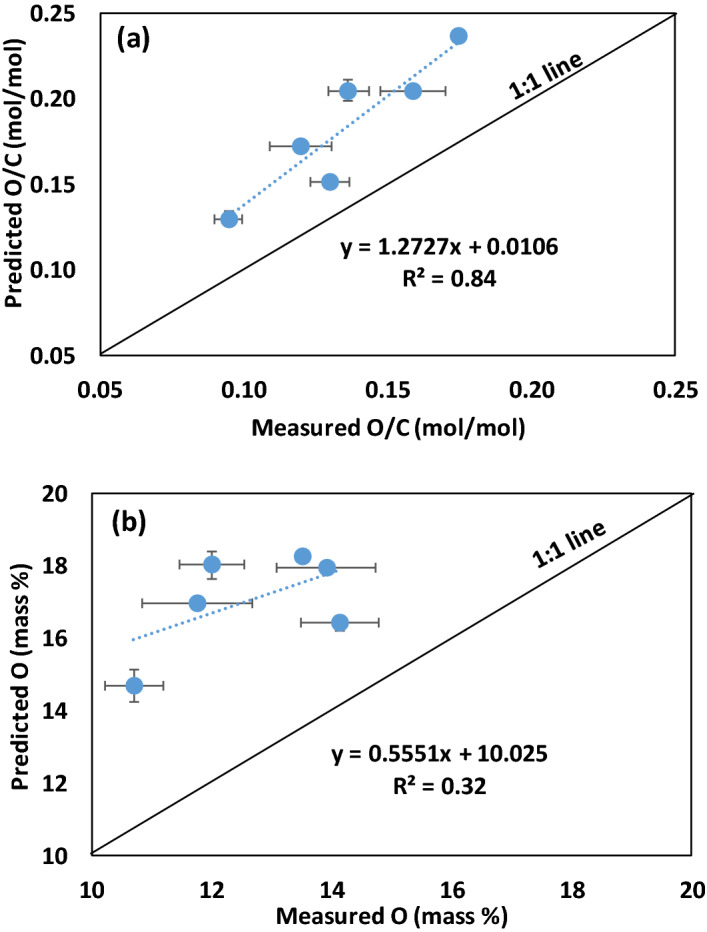
Relationships between measured and predicted a) O/C molar ratios and b) mass % organic O (oO) in untreated biochars produce from pyrolysis of untreated biomass feedstocks at 500 °C. Measured O (mass %) was determined by the conventional difference method, measured O/C molar ratios were determined by dividing %oO by %C and adjusting for atomic weights, predicted O/C molar ratios were determined using Eq. 10, and predicted oO (mass %) was determined using Eq. 11. Symbols showing standard error for sample size, n = 3.

The relationships between measure and predicted O/C molar ratios and mass %oO for the biochars produce from pyrolysis of untreated biomass feedstocks at 500 °C are shown in Fig. 5. The predicted O/C molar ratios and mass %oO were determined using our model (Eqs. 10 and 11); while the measured values were determined using the conventional difference method (where %oO = 100 - %C - %H - %N - %S - %Ash). There is a linear relationship between measured and predicted O/C molar ratios (R^2^ = 0.84) but no apparent relationship between measured and predicted %oO (R^2^ = 0.32) for the untreated biochars (Fig. 5). We attribute the discrepancy between the measured and predicted values to errors introduced by the use of the conventional difference method for determining the “measured” values. As discussed previously the ash prepared by ashing the biochars in a muffle furnace contains newly formed carbonates that were generated during the ashing procedure. These new carbonates did not exist in the biochar samples before the ashing procedure and therefore when the measured % ash is subtracted from 100 in the conventional difference method it effectively discounts some of the oO that existed in the biochar before ashing. Hence, we conclude that the conventional difference method seriously underestimates the %oO in the biochar samples.

For both O/C molar ratios and %oO, the predicted values were 19 to 53% higher than the measured values and the magnitude of the discrepancy was related to the ash content of the biochar samples. For example, the %oO content of SG biochar, which has 17.3% ash (determined by ashing in a muffle furnace) is 12.0% when determined by the conventional difference method and 18.5% when determined using Eq. 11. By comparison, the %oO content of RO biochar, which has only 1.1% ash, is 14.1% and 16.8% when determined by the difference method and Eq. 11, respectively. Herbaceous biochars, such as SG, contain large amounts of SiO_2_, the stoichiometry of which does not change when the biochar is ashed. By comparison, woody biochars, such as RO, contain only small amounts of SiO_2_. Although woody biochars contain much less total ash than herbaceous biochars, most of the ash in woody biochars is alkali and alkaline earth metals carbonates, which are newly formed during the ashing process. Thus, the magnitude of the discrepancy between the measured and predicted %oO of the biochars is influenced by the ash content of the biochars, however this is not a simple linear function.

**Figure 6:**
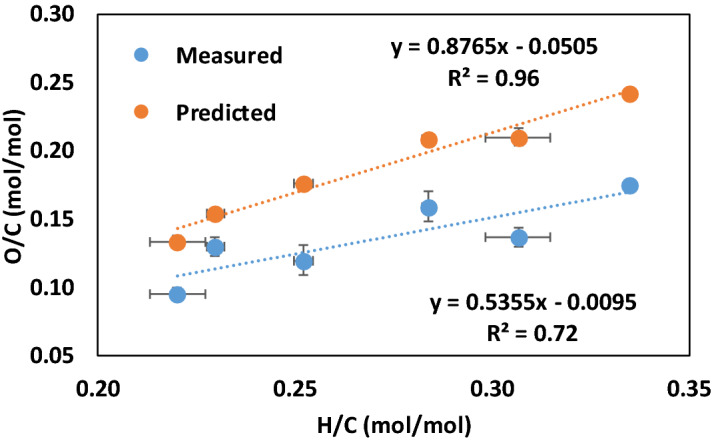
Relationship between O/C and H/C molar ratios for the untreated biomass biochars. %oO was determined by the conventional difference method for the “measured” and by Eq. 10 for the “predicted” O/C molar ratios. The results demonstrate that the accuracy of van Krevelen diagrams is adversely affected by use of the conventional difference method to determine %oO. Symbols showing standard error for sample size, n = 3.

The errors introduced by using the conventional difference method to determine %oO in biochars also adversely impacts van Krevelen diagrams, which have been widely used to assess the quality and stability of biochars^35^. Our data (Fig. 6) shows a substantial difference in the H/C vs. O/C molar ratio relationship when O/C molar ratios for biochars are determined using the conventional difference method to determine %oO (slope = 0.535, confidence interval range 0.92 at 95% confidence interval and R^2^ = 0.72, *P* < 0.05) vs. when O/C molar ratios are determined using Eq. 10 (slope = 0.876, confidence interval range 0.49 at 95% confidence interval and R^2^ = 0.96, *P* < 0.05). These results suggest that van Krevelen diagrams are sensitive to errors introduced by the conventional difference method and that much of the scatter in published van Krevelen plots^35–37^ may be due to these errors.

Untreated biomass biochars may contain some carbonate and bicarbonate anions before they are ashed, as such some of the C determined for the untreated biochars may have been inorganic C rather than organic C. The presence of significant amounts of inorganic C in a biochar sample would adversely affect the accuracy of both the O/C and H/C molar ratios. Fidel et al*.*^24^ quantified alkalis in various biochar samples including two of the same samples used in this study, corn stover and red oak biochars produced by pyrolysis at 500 °C. They report that the maximum amount of CO_2_ evolved from the corn stover biochar during acid treatment was 0.43 mmol g^−1^ and that the C content of the untreated corn stover biochar was 0.60 g g^-1^. Based on the results of Fidel et al.^24^ we estimate that the amount of inorganic C was less than 1% of the total C in the untreated biochars; and hence the impact of inorganic C on the accuracy of our model (Eqs. 10 and 11) was negligible. However, biochars produced from high ash feedstock, such as animal manure, may contain large amounts of inorganic C, which could adversely affect the accuracy of our model.

The %oO of biochars indicates the concentration of the O-containing functional groups and surface hydrophilicity and hydrophobicity^42^, and the O/C molar ratios of the organic phase of a biochar is an indicator of the level of biochar oxidation and biochar stability. Higher organic phase O/C molar ratios indicate greater levels of oxidation and lower biochar stability and therefore have the potential to be used in mechanistic models that predict agronomic and environmental responses to soil biochar applications^43^. Our results, however, have shown that it is difficult to accurately determine the %oO and molar O/C ratios of the organic phase. Wherever possible H/C molar ratios, which are intrinsically more accurate than O/C molar ratios, should be used as an index of biochar stability in the environmental modeling rather than O/C molar ratios.

## Conclusion

Estimates of %oO in biochar by the conventional difference method are not accurate because of iO in the ash. Acid washing of biochar removes most of the ash but also removes ~ 17% of the oO. We developed a model for estimating %oO and O/C molar ratios of fresh biochars. The model would need to be recalibrated to estimate %oO and O/C molar ratios for other post-pyrolysis treated (oxidation and weathering) biochars. We also developed a modified conventional difference method, in which %Si and %iO (estimated assuming SiO_2_ stoichiometry) replaced %ash to estimate the %oO content of acid washed biochars. Because of errors inherent in estimating the %oO in biochar O/C molar ratios and these errors can affect the slope and accuracy of van Krevelen plots. Authors are advised to use H/C molar ratios as indices of biochar aromaticity and stability.

## Methods

### Preparation of pure compound biochars

We measured the ash content of various pure compounds and selected 5 compounds with ash contents lower than 0.15%. These 5 compounds are cellulose (ash = 0.08%), polyethylene glycol (ash =  < 0.05%), vitamin C (ascorbic acid) (ash = 0.13%), glycine (ash = 0.07%) and tetracycline (ash =  < 0.01%). Commercially available cellulose and glycine feedstocks were purchased from Sigma-Aldrich, tetracycline was purchased from Fisher Scientific, and polyethylene glycol (MiraLAX) and vitamin C were purchased from the local store. Biochars were prepared from these 5 compounds using slow pyrolysis at various temperatures; 300, 400, 500, 600, 700, and 900 °C. All feedstocks were grounded using a mortar and pestle and oven dried at 65 °C for 2–3 d before use. A known amount of feedstock was transferred into pre-weighed ceramic crucibles, which were placed inside of a stainless-steel box with a N_2_ purge line in a muffle furnace. The muffle furnace was slowly heated to different peak temperatures, held at the peak temperature for one hour, and then cooled with continuous N_2_ purge (200 mL min^-1^) of the stainless-steel box. Finally, the biochars were grounded and sieved through 0.5 mm sieve and stored in closed containers prior to use. The mass of biochar produced was determined gravimetrically and the mass loss during pyrolysis was determined by difference from the mass of the starting feedstock. All analyses were done in triplicates.

### Characterization of pure compounds and biochars

The moisture content, volatile matter (VM), fixed carbon (FC) and ash content of the pure compound biochars were measured using the modified proximate analysis procedure^40^. Ultimate analysis (elemental C, H, N, and S) of the pure compounds and pure compound biochars was measured using a CNHS combustion analyzer (Vario Microcube, Elementar Analysensysteme GmbH, Germany), after biochar samples were ground in a ball mill. The O content of the pure compound biochars was calculated by subtracting total C, H, N, and S contents from unity (the conventional difference method). We calculated the mass recovery of C, H, N, S, and O using the measured elemental compositions of the biochars and pure compounds and the mass yield of biochar for each temperature. The mass loss of C, H, N, S, and O during biochar preparation was determined by difference. We consider these calculations to be valid because no other elements were present in significant quantities in the pure compounds or biochars produced from them. As a check of our procedure, we compared the theoretical O content of the pure compounds with the O content estimated by subtracting the sum of C, H, N, and S determined by thermal combustion, from unity and obtained excellent agreement (R^2^ = 0.97, *P* < 0.05).

### Types, sources and preparation of biochars from biomass feedstocks

A total of 6 biomass feedstocks, including 2 woody and 4 herbaceous feedstocks, were chosen for this study. The feedstocks were red oak (RO), loblolly pine (LP), switchgrass (SG), corn stover (CS), alfalfa meal (AM), and soybean stover (SS). Biochars were prepared using slow pyrolysis at 500 °C under constant N_2_ purging of 200 mL min^-1^, and then held at the peak pyrolysis temperature for one hour (the same method as described above for the pure compound biochars). Finally, biochars were grounded and sieved through 0.5 mm sieve and stored in closed containers prior to any analysis. The mass yield of the biomass biochars was determined gravimetrically and the mass lost during pyrolysis was calculated by difference from the starting biomass feedstock weight. Samples of these untreated biochars were saved for further analysis. Other samples of these biochars were treated with 0.05 M HCl to remove ash components and alkali and alkaline earth metals following the method of Fidel et al.^44^ (except the final CaCl_2_ wash step was omitted). The biochar yield after acid washing was calculated as follows: acid-washed biochar yield (%) = biochar yield (%) × fraction of biochar retained after acid washing. All measurements were done in triplicates.

### Characterization of acid treated and untreated biomass biochars

The moisture content, volatile matter (VM), fixed carbon (FC) and ash content of the untreated and acid treated biomass biochars were measured using the modified proximate analysis procedure^40^. Ultimate analysis (elemental C, H, N, and S) of biomass feedstocks and their biochars were measured using a CHNS combustion analyzer (Vario Microcube, Elementar Analysensysteme GmbH, Germany), after the biochar samples were ground in a ball mill. The untreated and acid treated biochars were ashed by heating them in air at 730 °C for 7–8 h, and C, H, N, and S content of the ash was determined using the same CHNS combustion analyzer. Inorganic elements in the ash from both untreated and acid treated biochars were determined by first solubilizing the ash in a mixture of HF and aqua regia in closed digestion vessels^45^, and then using an ICP-OES for elemental analysis following USEPA method 200.7^46^. We calculated the organic O content of the acid treated biochars by subtracting the sum of C, H, N, S, Si, and inorganic O (assuming SiO_2_ stoichiometry), from the mass of acid washed biochars (the modified difference method).

### Statistical analysis

All data sets were expressed as an arithmetic mean of three replicates (sample replications) with standard errors. Analysis of variance (ANOVA) was carried out using JMP 9.0.2 (SAS INSTITUTE, 2010). Variance homogeneity and least significant difference between different pure compound biochars and untreated and acid treated biochars were conducted. Slope test of two regression lines were compared based on the confidence intervals of the slopes. Statistical significance was accepted at the *P* < 0.05 level.

## Supplementary information

Supplementary figures.

## References

[1] Haberstroh PR (2006). Chemical composition of the graphitic black carbon fraction in riverine and marine sediments at sub-micron scales using carbon x-ray spectromicroscopy. Geochim. Cosmochim. Acta.

[2] Laird DA (2008). The charcoal vision: a win-win-win scenario for simultaneously producing bioenergy, permanently sequestering carbon, while improving soil and water quality. Agron. J..

[3] Masiello CA, Druffel ERM (1998). Black carbon in deep-sea sediments. Science (80-).

[4] Masiello CA, Druffel ERM, Currie LA (2002). Radiocarbon measurements of black carbon in aerosols and ocean sediments. Geochim. Cosmochim. Acta.

[5] Amonette, J. & Joseph, S. Characteristics of biochar: micro-chemical properties. In *Biochar for Environmental Management* (eds. Lehmann, J. & Joseph, S.) 35–54 (2009).

[6] Harvey OR, Herbert BE, Rhue RD, Kuo LJ (2011). Metal interactions at the biochar-water interface: energetics and structure-sorption relationships elucidated by flow adsorption microcalorimetry. Environ. Sci. Technol..

[7] Uchimiya M, Wartelle LH, Klasson KT, Fortier CA, Lima IM (2011). Influence of pyrolysis temperature on biochar property and function as a heavy metal sorbent in soil. Agric. Food Chem..

[8] Demirbas A (2004). Effects of temperature and particle size on bio-char yield from pyrolysis of agricultural residues. J. Anal. Appl. Pyro..

[9] Cheng CH, Lehmann J, Thies JE, Burton SD, Engelhard MH (2006). Oxidation of black carbon by biotic and abiotic processes. Org. Geochem..

[10] Crombie K, Masek O, Sohi SP, Brownsort P, Cross A (2013). The effect of pyrolysis conditions on biochar stability as determined by three methods. Glob. Chang. Biol. Bioenergy.

[11] Bakshi S, Aller DM, Laird DA, Chintala R (2016). Comparison of the physical and chemical properties of laboratory and field-aged biochars. J. Environ. Qual..

[12] Masiello CA (2004). New directions in black carbon organic geochemistry. Mar. Chem..

[13] Spokas KA (2010). Review of the stability of biochar in soils: predictability of O: C molar ratios. Carbon Manag..

[14] Budai, A. *et al. Biochar Carbon Stability Test Method: An Assessment of Methods to Determine Biochar Carbon Stability* (International Biochar Initiative, 2013).

[15] ASTM. *Method D3176-89. Standard Practice for Ultimate Analysis of Coal and Coke* (ASTM International, 2002).

[16] ASTM. D1762-84, Standard test method for chemical analysis of wood charcoal (2007).

[17] Domingues RR (2017). Properties of biochar derived from wood and high-nutrient biomasses with the aim of agronomic and environmental benefits. PLoS ONE.

[18] Bach Q-V, Chen W-H, Chu Y-S, Skreiberg Ø (2016). Predictions of biochar yield and elemental composition during torrefaction of forest residues. Biores. Technol..

[19] Wang S (2015). Physicochemical and sorptive properties of biochars derived from woody and herbaceous biomass. Chemosphere.

[20] Qiu, M. *et al.* Properties of the plant- and manure-derived biochars and their sorption of dibutyl phthalate and phenanthrene. *Sci. Rep.* **4**, **Article** (2014). 10.1038/srep05295 PMC4055907 24924925

[21] Peterson SC, Appell M, Jackson MA, Boateng AA (2013). Comparing corn stover and switchgrass biochar: Characterization and sorption properties. J. Agric. Sci..

[22] Enders A, Lehmann J (2012). Comparison of wet-digestion and dry-ashing methods for total elemental analysis of biochar. Commun. Soil Sci. Plant Anal..

[23] Bakshi S, Banik C, Laird DL (2018). Quantification and characterization of chemically-and thermally-labile and recalcitrant biochar fractions. Chemosphere.

[24] Fidel RB, Laird DA, Thompson ML, Lawrinenko M (2017). Characterization and quantification of biochar alkalinity. Chemosphere.

[25] Ronsse F, Van Hecke S, Dickinson D, Prins W (2013). Production and characterization of slow pyrolysis biochar: influence of feedstock type and pyrolysis conditions. Glob. Chang. Biol. Bioenergy.

[26] Lawrinenko M, Laird DA (2015). Anion exchange capacity of biochar. Green Chem..

[27] Gai X (2014). Effects of feedstock and pyrolysis temperature on biochar adsorption of ammonium and nitrate. PLoS ONE.

[28] Jindo K, Mizumoto H, Sawada Y, Sanchez-Monedero MA, Sonoki T (2014). Physical and chemical characterization of biochars derived from different agricultural residues. Biogeosciences.

[29] Wang Y, Hu Y, Zhao X, Wang S, Xing G (2013). Comparisons of Biochar Properties from Wood Material and Crop Residues at Different Temperatures and Residence Times. Energy Fuels.

[30] Baldock JA, Smernik RJ (2002). Chemical composition and bioavailability of thermally altered Pinus resinosa (Red pine) wood. Org. Geochem..

[31] Chen B, Zhou D, Zhu L (2008). Transitional adsorption and partition of nonpolar and polar aromatic contaminants by biochars of pine needles with different pyrolytic temperatures. Environ. Sci. Tech..

[32] Fang Q, Chen B, Lin Y, Guan Y (2014). Aromatic and hydrophobic surfaces of wood-derived biochar enhance perchlorate adsorption via hydrogen bonding to oxygen-containing organic groups. Environ. Sci. Tech..

[33] Keiluweit M, Nico PS, Johnson MG, Kleber M (2010). Dynamic molecular structure of plant biomass-derived black carbon (biochar). Environ. Sci. Technol..

[34] Xiao X, Chen Z, Chen B (2016). H/C atomic ratio as a smart linkage between pyrolytic temperatures, aromatic clusters and sorption properties of biochars derived from diverse precursory materials. Sci. Rep..

[35] Brewer CE (2012). Extent of pyrolysis impacts on fast pyrolysis biochar properties. J. Environ. Qual..

[36] Hammes K (2006). Synthesis and characterisation of laboratory charred grass straw (*Oryza sativa*) and chestnut wood (*Castanea sativa*) as reference materials for black carbon quantification. Org. Geochem..

[37] van Krevelen, D. W. *Coal Science* (1957).

[38] Ouellette, R. J. & Rawn, J. D. 20 - Carboxylic Acids. In (eds. Ouellette, R. J. & Rawn, J. D. B. T.-O. C.) 659–698 (Elsevier, 2014). 10.1016/B978-0-12-800780-8.00020-6

[39] Lupoi JS, Smith EA (2012). Characterization of woody and herbaceous biomasses lignin composition with 1064 nm dispersive multichannel Raman spectroscopy. Appl. Spectrosc..

[40] Aller D, Bakshi S, Laird DA (2017). Modified method for proximate analysis of biochars. J. Anal. Appl. Pyro..

[41] Kim P (2011). Surface functionality and carbon structures in lignocellulosic-derived biochars produced by fast pyrolysis. Energy Fuels.

[42] Schimmelpfennig S, Glaser B (2012). One step forward toward characterization: some important material properties to distinguish biochars. J. Environ. Qual..

[43] Archontoulis SV (2015). A model for mechanistic and system assessments of biochar effects on soils and crops and trade-offs. Glob. Chang. Biol. Bioenergy.

[44] Fidel RB, Laird DA, Thompson ML (2013). Evaluation of modified Boehm titration methods for use with biochars. J. Environ. Qual..

[45] Hossner, L. R. Dissolution for total elemental analysis. In *Methods of Soil Analysis: Part 3- Chemical Methods* (ed. Sparks, D. L.) 49–64 (Soil Sci. Soc. Am., 1996).

[46] USEPA. Method 200.7: Determination of metals and trace elements in water and wastes by inductively coupled plasma-atomic emission spectrometry (2007).

