# Supplementary material for: Estimating the organic oxygen content of biochar
Source: Sci Rep. 2020 Aug 4;10:13082. doi: 10.1038/s41598-020-69798-y (PMC7403298; doi:10.1038/s41598-020-69798-y)
Supplement: Supplementary file 1 — Supplementary figures. [file 41598_2020_69798_MOESM1_ESM.docx]

**Supporting Information**

Title: Estimating the organic oxygen content of biochar

Author/s: Santanu Bakshi, Chumki Banik and David A. Laird

This supporting information contains 3 figures.

Corresponding author:

Santanu Bakshi

3108 Biorenewable Research Laboratory, Bioeconomy Institute, Iowa State University,

Ames, IA 50011, USA

Tel: +1-515-294-4984; email: [santanubakshi@gmail.com](mailto:santanubakshi@gmail.com)

Figure S1: Effect of peak pyrolysis temperature on mass yield of biochars produced by slow pyrolysis from pure compounds.

Figure S2: Effect of peak pyrolysis temperature on (a) carbon, (b) oxygen, and (c) hydrogen content of biochars produced by slow pyrolysis from pure compounds.

Figure S3: Effect of peak pyrolysis temperature on (a) H/C and (b) O/C molar ratios of biochars produced by slow pyrolysis from pure compounds.
